# Effects of baicalin on collagen Ι and collagen ΙΙΙ expression in pulmonary arteries of rats with hypoxic pulmonary hypertension

**DOI:** 10.3892/ijmm.2015.2110

**Published:** 2015-02-25

**Authors:** PANPAN LIU, SHUANGQUAN YAN, MAYUN CHEN, ALI CHEN, DAN YAO, XIAOMEI XU, XUEDING CAI, LIANGXING WANG, XIAOYING HUANG

**Affiliations:** 1 Intensive Care Unit, Ningbo Medical Treatment Center Lihuili Hospital, Ningbo, Zhejiang 315040, P.R. China; 2 Division of Respiratory Medicine, Taizhou Enze Medical Center Luqiao Hospital, Taizhou, Zhejiang 318050, P.R. China; 3 Division of Pulmonary Medicine, The First Affiliated Hospital of Wenzhou Medical University and Key Laboratory of Heart and Lung, Wenzhou, Zhejiang 325035, P.R. China

**Keywords:** baicalin, hypoxia, pulmonary hypertension, collagen, remodeling of the pulmonary artery, a disintegrin and metalloprotease with thrombospondin type-1 motif

## Abstract

The synthesis and accumulation of collagen play an important role in the formation and progression of hypoxic pulmonary hypertension. Baicalin has been reported to prevent bleomycin-induced pulmonary fibrosis. However, the role of baicalin in the treatment of pulmonary hypertension remains unknown. A disintegrin and metalloprotease with thrombospondin type-1 motif (ADAMTS-1) is a secreted enzyme that acts on a wide variety of extracellular matrix (ECM) substrates associated with vascular diseases. In this study, we aimed to investigate the effects of baicalin on the synthesis of collagen I in rats with pulmonary hypertension induced by hypoxia and the changes in ADAMTS-1 expression. A total of 24 Sprague Dawley rats were randomly assigned to 3 groups as follows: the control group (C), the hypoxia group (H) and the hypoxia + baicalin group (B). The rats in groups H and B were kept in a normobaric hypoxic chamber for 4 weeks, and the rats in group C were exposed to room air. We measured the hemodynamic indexes, including mean pulmonary artery pressure (mPAP), mean systemic (carotid) artery pressure (mSAP), and then calculated the mass ratio of right ventricle to left ventricle plus septum [RV/(LV + S)] to reflect the extent of right ventricular hypertrophy. We measured the mRNA and protein expression levels of type I collagen, type III collagen and ADAMTS-1 by hybridization *in situ*, and immunohistochemistry and western blot analysis, respectively. The results revealed that treatment with baicalin significantly reduced pulmonary artery pressure and attenuated the remodeling of the pulmonary artery under hypoxic conditions by increasing the expression of ADAMTS-1, so that the synthesis of type I collagen and its mRNA expression were inhibited. In conclusion, baicalin effectively inhibits the synthesis of collagen I in pulmonary arteries and this is associated with an increase in the expression of ADAMTS-1. Thus, treatment with baicalin may be an effective method for lowering pulmonary artery pressure and preventing pulmonary artery remodeling.

## Introduction

Pulmonary arterial hypertension (PAH) is a common and multifactorial disease characterized by the progressive remodeling of the small pulmonary arterial, and with the progression of the disease, this can finally lead to an elevation in pulmonary vascular resistance, right ventricular dysfunction and even death (1,2).

Wang *et al* reported that pulmonary arterial collagen accumulation plays an important role in hypoxic pulmonary hypertension (HPH)-induced pulmonary vascular remodeling in distal arteries and large proximal arteries (3). It can also prevent normal hemodynamic recovery which may have severe consequences for right ventricular function (4–6). In hypoxia-induced pulmonary vascular adventitial remodeling, Zhang *et al* (7) discovered that hypoxia increases the expression of type I collagen in cultured fibroblasts. Changes in the levels of extracellular matrix (ECM) proteins, particularly those of collagen have been proven to contribute to arterial stiffening, and the content of collagen in mice remains at a higher level even after being removed from the hypoxic environment due to impaired type I collagen degradation (4).

Baicalin is a flavonoid compound purified from the dry roots of *Scutellaria baicalensis*, which has been demonstrated to possess multiple pharmacological activities, including antioxidant, antitumor, anti-inflammatory and anti-proliferative activities (8,9). Baicalin has been shown to exert certain therapeutic effects on rats with hepatic fibrosis induced by carbon tetrachloride and to significantly attenuate the degree of hepatic fibrosis, and decrease the collagen area and the collagen area percentage in liver tissue (10). Baicalin has been demonstrated to have anti-fibrotic activity by suppressing collagen I expression at both the mRNA and protein level (11). Yet, there are only few reports concerning the effects of baicalin on hypoxia-induced pulmonary hypertension.

A disintegrin and metalloprotease with thrombospondin motif (ADAMTS) proteinases were first described in mice by Kuno *et al* in 1997 (12) and have subsequently been identified in mammals and *Caenorhabditis elegans*. Thus far, 19 distinctive human ADAMTS gene products have been identified. ADAMTS proteases are secreted enzymes that act on a wide variety of ECM substrates, including pro-collagen, proteoglycans, hyalectans and cartilage oligomeric matrix protein (13–16). It has been reported that the expression of ADAMTS-1 contributes to tissue destruction and has been implicated in vascular diseases, such as atherosclerosis. It may also destroy the aortic wall by degrading other ECM components (17–19). In addition, ADAMTS-1 levels in chronic viral myocarditis (CVMC) have been found to negatively correlate with type I collagen levels. Hence, it was concluded that ADAMTS-1 contributes to the anti-fibrotic effect by accelerating the degradation of type I collagen in CVMC (20).

Based on the possible links among HPH, collagen, baicalin and ADAMTS-1, in the present study, we created a rat model of HPH, and measured the expression levels of collagen and ADAMTS-1 in order to determine the effects of baicalin on the synthesis of collagen in rats with HPH.

## Materials and methods

### Materials

The polyclonal rabbit anti-rat type I collagen (ab34710) and type III collagen (ab7778) antibodies were obtained from Abcam (Cambridge, UK). The polyclonal goat anti-rabbit antibody (sc-2004) and polyclonal rabbit anti-rat ADAMTS-1 antibody (sc-25581) were purchased from Santa Cruz Biotechnology (Santa Cruz Biotechnology, Inc., Santa Cruz, CA, USA). Secondary HRP-linked goat anti-rabbit antibody (sv-0002) was obtained from Boster Biotech Co., Ltd. (Wuhan, China). Baicalin was obtained from Sigma-Aldrich (St. Louis, MO, USA). The hybridization kit *in situ* was purchased from Boster Biotech Co., Ltd. Adult male Sprague-Dawley (SD) rats (obtained from the Laboratory Animal Centre of Wenzhou Medical University, Wenzhou, China) weighing 250–300 g were used in our experiments in accordance with the guidelines for animal procedures provided by Wenzhou Medical College and the National Institutes of Health standards for animal care. An effort was made to reduce the number of experimental animals used and to minimize their suffering. This study was approved by the Animal Ethics Committee of Wenzhou Medical College under permit number SCXK (Shanghai 2010–0002).

### Treatment of animals

The animal models and experimental groups were as follows: 24 male SPF SD rats weighing 250–300 g provided by the Experimental Animal Centre of Wenzhou Medical University were randomly assigned to the following 3 groups (8 rats/group): the control group (C), the hypoxic group (H) and the hypoxia + baicalin group (B). The rats in groups H and B were kept in a normobaric hypoxic (80–110 ml/l O_2_, 8 h/day) chamber for 4 weeks. The rats in group C were exposed to room air. Anhydrous calcium chloride and sodium hydroxide were used for water and carbon dioxide absorption, respectively. Baicalin was injected intraperitoneally into the rats in group B at a daily dose of 30 mg/kg, 30 min before they were placed in the chamber.

### Measurement of hemodynamic indexes: mean pulmonary artery pressure (mPAP), mean systemic (carotid) artery pressure (mSAP) and mass ratio of right ventricle to left ventricle plus septum [RV/(LV + S)]

The rats were anesthetized by an intraperitoneal injection of 5% chloral hydrate (40 mg/kg body weight). A polyethylene catheter (inside diameter, 0.9 mm; outside diameter, 1.1 mm) was gradually inserted into the pulmonary artery through an incision in the right external jugular vein and the mPAP was recorded. The rats were then anatomised after blood samples were obtained. We collected and weighed the left ventricle plus the interventricular septum (LV + S) and the right ventricle (RV) tissue by cutting along the edge of the ventricle and the interventricular septum. The mass ratio of the RV to the (LV + S) was used as the index for RV hypertrophy.

*Hematoxylin and eosin staining* Following deparaffinization and dehydration, the sections of lungs were incubated in hematoxylin solusion for 10–30 min and then washed with running water for 15 min. The sections were then placed into 1% hydrochloric acid ethanol for 2–10 sec before being dehydrated by a graded ethanol series. Finally, they were dehydrated again immediately following counterstaining with 0.5% eosin solution for 1–3 min.

### Immunohistochemistry

Following blocking, the sections were incubated with rabbit anti-rat type I collagen polyclonal antibody [diluted 1:500 with phosphate-buffered saline (PBS)], type III collagen antibody (diluted 1:500 with PBS), ADAMTS-1 antibody (diluted 1:25 with PBS). They were then incubated in secondary HRP-linked goat anti-rabbit antibody. Lung specimens incubated with 10% goat serum in place of the specific primary antibody served as the negative controls. Immunoreactivity was visualized using 3,30-diaminobenzidine (DAB). All antibodies used for immunohistochemistry were diluted in PBS, and 5 fields of vision were selected randomly from each section for quantitative analyses. Using Image-Pro Plus software, we measured the area of tunica media of pulmonary arterioles and the integrated optical density of positive products, then we calculated the ratio of the integrated optical density and area and named the average optical density (OD value) to reflect the expression of positive products.

### Hybridization in situ

The sections of each case were deparaffinized and dehydrated through a graded ethanol series, then in 3% hydrogen peroxide solution for 10 min. Subsequently, each section was digested by pepsin (1.3 mg/ml; Dako, Glostrup, Denmark) for 30 min, washed in 0.1 M PBS (pH 7.4) buffer and fixed with 4% polyformaldehyde-PBS liquor for 10 min, then washed 3 times with PBS buffer for 5 min. The probes and tissue RNA were co-denatured at 40°C overnight. They were then washed with hybridization buffer (2X SSC for 5 min 2 times, 0.5X SSC and 0.2X SSC for 15 min only once, respectively). This was followed by incubation with blocking buffer at 37°C for 30 min, and the sections were checked with a digoxin reagent box. Subsequently, they were color-produced by chromogenic reagent and were monitored visually and this reaction was terminated by placing the slides in water. The slides were counterstained with nucleus fixed red reagent to visualize the nuclei, then washed again with water, dehydrated, vitrified by dimethylbenzene, and mounted with neuter balata. The negative control was hybridized with no-probe liquor in parallel with the experimental reactions and 5 fields of vision were selected randomly from each section for quantitative analyses.

### Western blot analysis

Fresh lung tissue weighing 50 mg was homogenized with a glass homogenizer on ice, then lysed with chilled lysis buffer followed by centrifugation at 12,000 × g for 30 min at 4°C. The supernatant was collected as total protein and transferred to a new cooled 1.5 ml Eppendorf tube. The protein concentration was measured by the Bradford method and the homogenate was diluted to 2 *μ*g/*μ*l with PBS. Equal amounts of proteins (20 *μ*g) were separated by 12% sodium dodecyl sulfate-polyacrylamide gel (SDS-PAGE) and transferred onto polyvinylidene fluoride (PVDF) membranes (Millipore, Billerica, MA, USA). The blots were blocked in Tris-buffered saline with Tween-20 (TBST) containing 5% bovine serum albumin (BSA) for 4 h. Subsequently, the membranes were incubated overnight at 4°C with specific primary antibodies: rabbit anti-rat collagen I antibody at a dilution of 1:1,000, rabbit anti-rat collagen III antibody at a dilution of 1:1,000. The membranes were incubated with peroxidase-labeled affinity purified antibody to rabbit IgG (0.1 *μ*g/ml) after washing with TBST buffer and visualized using an enhanced chemiluminescence kit (Pierce, Rockford, IL, USA) with an ECL Imaging System (Bio-Rad Laboratories, Hercules, CA, USA). Band intensities were quantified using Image-Pro Plus software.

### Statistical analysis

Values are presented as the means ± SD. All data were analyzed using one-way ANOVA followed by a LSD-test. A value of P<0.05 was considered to indicate a statistically significant difference between groups.

## Results

### Baicalin improves hemodynamics, attenuates right ventricular remodeling and morphological changes in pulmonary arterioles in rats with HPH

**Figure 1:**
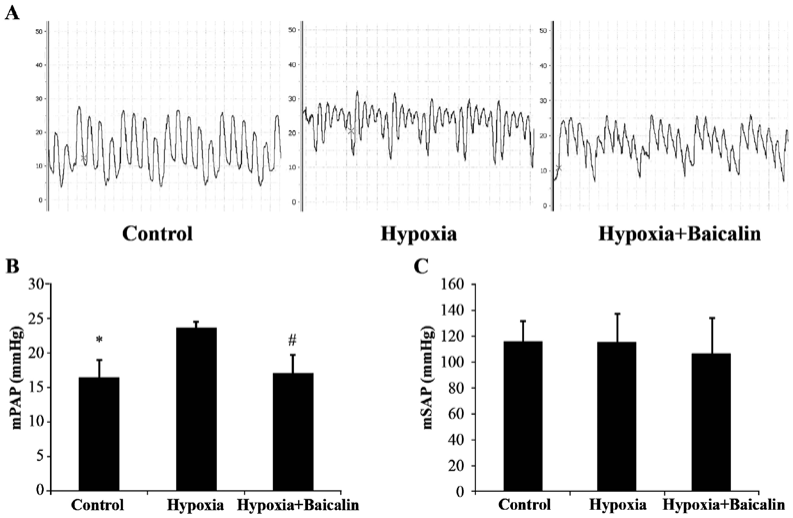
Effect of baicalin on hypoxia-induced pulmonary hypertension. (A) Demonstrative traces of mean pulmonary artery pressure (mPAP) in each group of animals. (B) mPAP. ^*^P<0.01, compared with hypoxia group; ^#^P<0.01, compared with hypoxia group. (C) Mean systemic (carotid) arterial pressure (mSAP).

**Figure 2:**
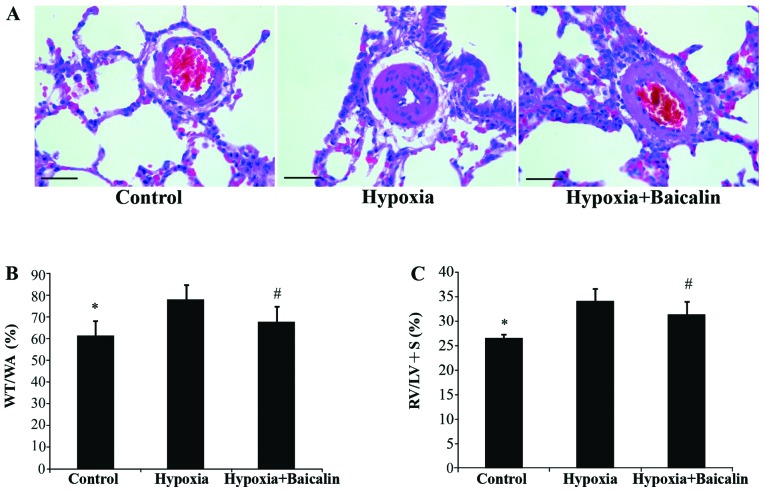
Effects of baicalin on pulmonary vascular remodeling and right ventricular hypertrophy. (A) Hematoxylin and eosin staining (scale bar, 50 *μ*m; magnification, x400) (B) The ratios of vessel thickness and media wall thickness [ratio of the pulmonary artery wall thickness to the total area of the artery (WT/WA)%]. ^*^P<0.01, compared with hypoxia group; ^#^P<0.01, compared with hypoxia group. (C) Right ventricular hypertrophy [mass ratio of right ventricle to left ventricle plus septum (RV/LV + S)]. ^*^P<0.01, compared with hypoxia group; ^#^P<0.05, compared with hypoxia group.

We measured mPAP and mSAP to reflect the hemodynamic changes. Hematoxylin and eosin (H&E) staining of the pulmonary artery tissue was carried out to calculate the ratio of the pulmonary artery wall thickness to the total area of the artery (WA/TA) for a comparison of the thickening of the arteries among the 3 groups. The RV/(LV + S) was calculated to reflect the extent of right ventricular hypertrophy. mPAP increased from 16.3±2.7 to 23.6±0.9 mmHg following the induction of chronic hypoxia (P<0.01), and treatment with baicalin effectively attenuated this increase, decreasing mPAP to 17.0±2.7 mmHg (P<0.01; Fig. 1A and B). However, there was no statistically significant difference in mSAP among the 3 groups (Fig. 1C). The thickness of the arteries which was induced by chronic hypoxia (77.75±6.79%) was much more obvious than that of the rats in group C (61.00±6.86%) (P<0.01). Bacalin markedly attenuated this effect, decreasing the WA/TA and the WA/TA% to 67.72±6.76% (P<0.01; Fig. 2A and B). The RV/(LV + S)% was markedly elevated in the rats in group H (34.18±2.43%) compared with the rats in group C (26.57±0.77%) (P<0.01). Following the injection of bacalin, the RV/(LV + S)% decreased to 31.36±2.70% and right ventricular hypertrophy was significantly attenuated (P<0.05; Fig. 2C).

### Baicalin inhibits the protein and mRNA expression of collagen I induced by chromic hypoxia

**Figure 3:**
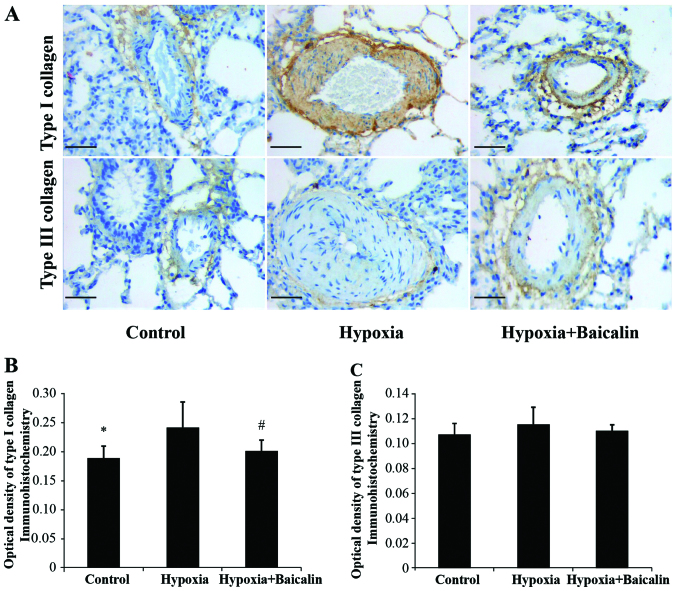
Protein expression of collagen I and III in pulmonary arterioles (by immunohistochemical analysis). (A) Expression of collagen I and III in pulmonary arterioles (scale bar, 50 *μ*m; magnification, x400). (B) Expression of collagen I in pulmonary arterioles. ^*^P<0.01, compared with hypoxia group; ^#^P<0.05, compared with hypoxia group. (C) Expression of collagen III in pulmonary arterioles.

**Figure 4:**
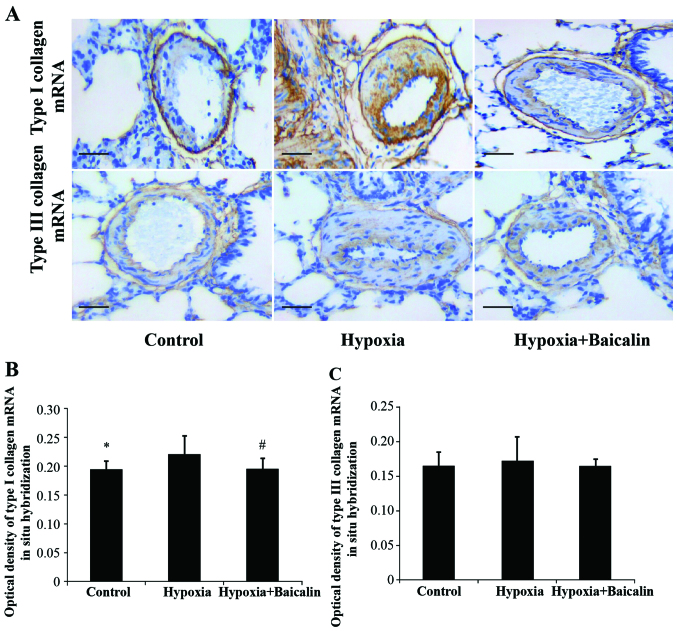
mRNA expression of collagen I and III in pulmonary arterioles (by hybridization *in situ*). (A) mRNA expression of collagen I and III in pulmonary arterioles (scale bar, 50 *μ*m; magnification, x400). (B) mRNA expression of collagen I in pulmonary arterioles ^*^P<0.05, compared with hypoxia group; ^#^P<0.05, compared with hypoxia group. (C) mRNA expression of collagen III in pulmonary arterioles.

**Figure 5:**
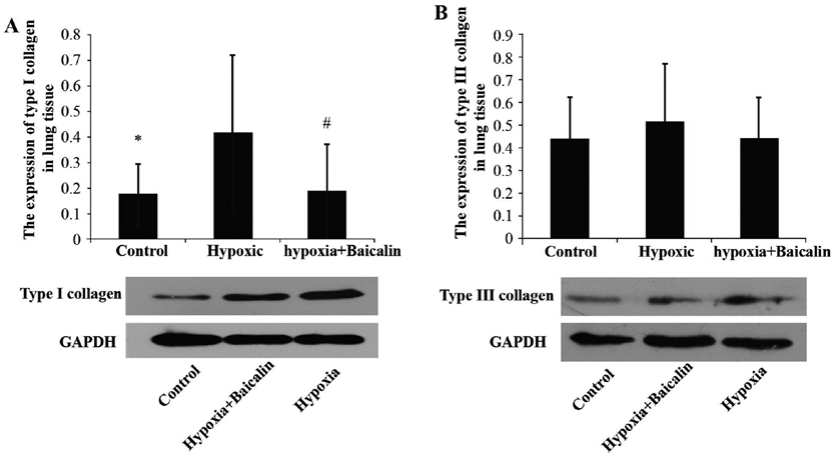
Protein expression of collagen I and III protein in lungs (by western blot analysis). (A) Protein expression of collagen I in lungs. ^*^P<0.05, compared with hypoxia group; ^#^P<0.05, compared with hypoxia group. (B) Protein expression of collagen III in lungs.

Under the condition of chronic hypoxia, the OD value of collagen I noticeably increased compared with the normal condition in group C (0.242±0.043 vs. 0.188±0.021, P<0.01), and its mRNA expression increased from 0.195±0.014 in group C to 0.220±0.033 in group H (Figs. 3 and 4). However, as expected, treatment with baicalin decreased the OD value and mRNA expression of collagen I to 0.201±0.019 (P<0.05) and 0.196±0.018 (P<0.05), respectively (Figs. 3 and 4). Western blot analysis also revealed that chronic hypoxia markedly upregulated the protein expression of collagen I from 0.175±0.119 to 0.417±0.305 (P<0.05), and treatment with baicalin markedly decreased this expression (0.188±0.183; P<0.05; Fig. 5).

### Baicalin has little effect on the protein and mRNA expression of collagen III in rats suffering from HPH

The protein expression of collagen III was detected by immunochemistry and western blot analysis. Hybridization *in situ* was carried out to determine the changes in mRNA expression. As shown in Figs. 3 and 4, there were no apparent statistically significant differences in the mRNA expression of collagen III among the 3 groups (P>0.05). A similar tendency was detected by western blot analysis (Fig. 5).

### Baicalin increases the expression of ADAMTS-1 in rats in response to chronic hypoxia

**Figure 6:**
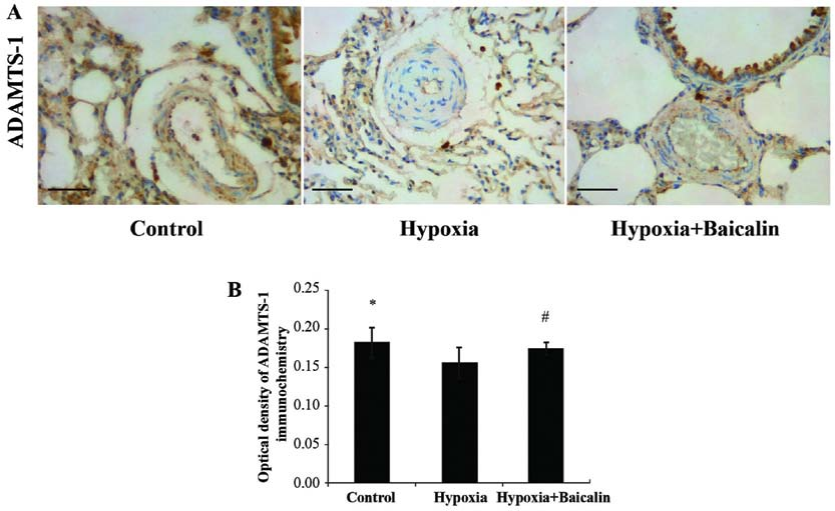
Protein expression of a disintegrin and metalloprotease with thrombospondin type-1 motif (ADAMTS-1) in pulmonary arterioles (by immunohistochemical analysis). (A) Expression of ADAMTS-1 in pulmonary arterioles (scale bar, 50 *μ*m; magnification, x400). (B) Protein expression of ADAMTS-1 was downregulated in hypoxia group and upregulated in hypoxia + baicalin group, ^*^P<0.01, compared with hypoxia group; ^#^P<0.05, compared with hypoxia group.

Immunohistochemistry was carried out to detect the protein expression of ADAMTS-1 in the rats suffering from PAH. As shown in Fig. 6, there was a marked decrease in ADAMTS-1 expression in the pulmonary arterioles of the rats under chronic hypoxic conditions (0.156±0.020) compared to normal conditions (group C) (0.182±0.020; P<0.01); however, treatment with baicalin significantly increased ADAMTS-1 expression in the rats in group B (0.174±0.009) compared to the rats in group H (P<0.05).

These results suggested that chronic hypoxia stimulated the synthesis and deposition of collagen I, which played a key role in the process of pulmonary vascular remodeling. Simultaneously, a decrease in ADAMTS-1 expression was observed. Baicalin was proven to effectively lower mPAP, which also reversed pulmonary vascular remodeling partially by upregulating the expression of ADAMTS-1, and thus inhibiting the expression of collagen I, further exerting protective effects on rats suffering from HPH.

## Discussion

PAH is a fatal disease which progresses rapidly with high mortality. HPH has been recognized as the most common complication of some pulmonary diseases characterized by the vasoconstriction and remodeling of the pulmonary vascular artery which has been confirmed to play a role in the development of HPH (21,22). Exposure to a hypoxic environment for 4 weeks can be used to successfully establish a model of chronic HPH (23). This study demonstrated that mPAP was significantly higher in the hypoxic group than in the control group. The thickening of the vascular wall and stenosis of the lumen were obvious changes in the morphology of the pulmonary arterioles. After calculating the RV/(LV + S) ratio as the index of right ventricular hypertrophy and the WA/TA%, we found that our data not only were consistent with those of our previous studies, but also indicated success in establishing the model of HPH (24,25).

In recent years, increasing attention has been paid to the association between HPH and collagen accumulation. Studies have discovered that collagen accumulation plays a key role in the stiffening of the proximal pulmonary artery, which leads to sustained increase in pulmonary artery pressure and finally the failure of the right ventricle (3,26,30). Research on newborn Wistar rats exposed to hypoxia has also revealed increased mPAP, right ventricular hypertrophy, collagen deposition in the ECM and pulmonary vascular remodeling (26). In the study by Wang *et al*, it was suggested that the collagen total content was critical to extralobar pulmonary artery stiffening during HPH (27). Type I collagen is a fibrillar collagen subtype that plays a dominant role in the composition and strength of the arteries (28–30). The expression of collagen, including collagen I in rats exposed to hypoxia has been shown to markedly increase pulmonary vascular remodeling. This increase may be alleviated by inhibiting the collagen accumulation in pulmonary arteries (31). In this study, we also demonstrated a significantly increased protein and mRNA expression of collagen I in the pulmonary arterial wall accompanied by the stenosis of the lumen and the thickening of the pulmonary arterial wall under hypoxic conditions, contributing to the formation and progression of pulmonary vascular remodeling, which is in accordance with what was observed in our previous study (25).

Baicalin is a plant-derived flavonoid with a variety of activities, including antioxidant and anti-inflammatory properties, and is also known to alleviate ischemia-reperfusion injury (8,9). It has been previoulsy demonstrated that baicalin is essential for mesenchymal stem cell (MSC) transplantation, exerting a therapeutic effect by reducing the fibrotic area (32). Huang *et al* (34) found that baicalin attenuated human pulmonary artery smooth muscle cell proliferation and the phenotypic switch induced by transforming growth factor-β1. It has also been shown to prevent bleomycin-induced pulmonary fibrosis in rats, and that the above effect of baicalin is related to the blockage of the synthesis of type I collagen and the decrease in the number of myofibroblasts in the lungs (33,34). However, studies on its effects on HPH are limited. In this study, baicalin was found to be effective in lowering mPAP and the expression of collagen I in the pulmonary artery, thus partially reversing pulmonary vascular remodeling, which may be a critical mechanism through which baicalin protects rats with HPH.

Kaushal and Shah (35) previously demonstrated that proteolytically active ADAMTS-1 participates in normal ECM turnover and that its absence contributes to the fibrosis observed in mutant mice. The upregulation of ADAMTS (ADAMTS-1, -4 and -15) has been reported to be involved in disc ECM destruction in the human degenerated intervertebral disc (36). Rehn *et al* found that ADAMTS-1 induced collagen type I processing in bone *in vitro* together with a positive influence on osteoblastic three-dimensional growth by promoting collagen degradation directly or indirectly (37). In this study, we demonstrated that in rats with HPH, the expression of ADAMTS-1 in the pulmonary arterioles decreased accompanied by an increase in the expression of collagen I and not collagen III. Following treatment with baicalin, there was a significant increase in ADAMTS-1 expression and a decreased in collagen I expression. We also observed a marked improvement in hemodynamics and in the morphology of the pulmonary arterioles.

The results of the present study indicate that the ADAMTS-1 participates in the inhibition of the synthesis of collagen I by baicalin, suggesting that baicalin exerts protective effects on rats with HPH and reserves pulmonary vascular remodeling partially by increasing ADAMTS-1 expression, thus inhibiting the overexpression of collagen I.

Taken together, our novel (to the very best of our knowledge) findings suggest that ADAMTS-1 is involved in the development of HPH, and that baicalin upregulates the expression of ADAMTS-1 under chronic hypoxic conditions, thus inhibiting the synthesis and expression of collagen I, contributing to the decrease in pulmonary hypertension and pulmonary vessel remodeling.
